# What factors affecting investment decision? The moderating role of fintech self-efficacy

**DOI:** 10.1371/journal.pone.0299004

**Published:** 2024-04-18

**Authors:** Norhazimah Che Hassan, Aisyah Abdul-Rahman, Siti Ngayesah Ab. Hamid, Syajarul Imna Mohd Amin

**Affiliations:** 1 Ministry of Finance Malaysia, Putrajaya, Malaysia; 2 Faculty of Economics and Management & Institute of Islam Hadhari, Universiti Kebangsaan Malaysia, Bangi, Malaysia; 3 Faculty of Economics and Management, Universiti Kebangsaan Malaysia, Bangi, Malaysia; Universiti Teknologi MARA, MALAYSIA

## Abstract

This study aims to determine, from the perspective of investors, the factors that predict Islamic unit trust (IUT) investment intentions. Additionally, this paper examines the moderating effect of fintech self-efficacy (FSE) on the relationship between attitude and investment intention. A total of 392 data were collected from IUT investors in Malaysia and analyzed using partial least squares structural equation modeling. The findings reveal that subjective norms have the highest impact on investment intention, followed by attitude and FSE, while religiosity is not significantly associated with investment intention in Islamic unit trust funds. Attitude significantly mediates religiosity-intention and Islamic financial literacy-intention relationships. FSE significantly moderates the attitude-intention relationship. The results shed light on the key factors that increase investing behavior and have direct managerial implications with regard to marketing strategies and target markets. These findings suggest that IUT service providers should take the lead in attracting customers through effective and targeted marketing initiatives, particularly by enhancing customers’ FSE and capabilities. This study provides empirical evidence on the interrelationships between Islamic financial literacy, religiosity, and FSE in examining investors’ behavior using the Theory of Planned Behavior framework. The study explores the moderating role of FSE on the relationship between attitude and investment intention.

## Introduction

A unit trust fund is a collection of funds invested by individuals with similar investment and financial goals, professionally managed by a qualified fund manager [1]. Unit trust funds provide small investors and families with economic participation in commercial channels such as securities, bonds, and other financial instruments. It has been an expanding segment of the capital market for decades. Risk management and analysis of investor variables are crucial for market operations. Small investors with limited knowledge, ability, and risk tolerance can also invest in profitable portfolios managed by more qualified fund managers through unit trusts. To maximize client returns, these technical administrators target profitable and high-performing financial products. Unit trust is one of the most common investment vehicles used by investors to generate capital, and Muslims, in particular, would choose Islamic unit trust funds for their investments because they align with Islamic principles. Islamic unit trust funds differ from conventional funds in the sense that these funds involve several important matters such as fund objectives, investment strategies, operations and fund management, documentation including contracts, channels and activities, accounts and reporting, and returns, all of which must comply with the principles outlined by Shariah [2].

Malaysia is renowned as a pioneer and leader in the development of Islamic finance [3]. It is one of the prominent global hubs for the Islamic capital market (ICM) [4]. It is also one of the concentration markets for Islamic funds’ assets in the form of equity, fixed-income, sukuk, money market, and real estate [5]. However, the demand for Islamic unit trust funds (IUT) is distinctively small relative to conventional funds. Out of RM526.89 billion NAV of unit trusts, only RM128.33 billion (24.4%) is Shariah-compliant, with 3.2 million (15.7%) unit trust holder accounts compared to the 17.3 million (84.6%) conventional ones [4]. Thus, it demonstrates massive potential growth of the IUT market to penetrate the untapped investors in Malaysia, considering it is a Muslim-majority country and internationally, with a prediction that the global Muslim population to reach 2.2 billion by 2030 [6].

The surge in the global Muslim population who practice Islam as a way of life has created the demand for Shariah-compliant investment that complies with Islamic tenets. Undeniably, IUT offers a viable investment alternative to conventional funds. It recently satisfied non-Muslim communities worldwide attracted to Shariah values that promote ethics, justice, and generosity [7]. Past research has claimed that IUT is a safe-haven investment, with lower risk, less drawdown by investors, better resilient, and faster recovery during economic uncertainty such as the 2008 financial crisis and the Covid-19 pandemic [8, 9]. Besides, the mutual fund industry has more potential to grow in line with financial technology (Fintech) development [10]. Fintech is revolutionizing the financial industry at an unprecedented rate [11], from mobile payments, robo-advisor platforms, and application-based investments, to online banking solutions [12]. The IUT is no exception, and fintech automates the system of finance and investment, operations and risk management, and data security [13]. Nonetheless, the unsolved puzzle remained, why is IUT less appealing among investors? How can more investors be attracted to invest in IUT? What factors influence investors’ decisions in IUT? Therefore, it is vital to understand the factors influencing investors’ investment intentions in IUT to promote the development of the IUT industry.

Numerous studies have examined investment behavioral intention but neglected IUT. For example, several studies have investigated stocks [14, 15], bonds [16, 17], and retirement fund investment schemes [18]. Although most studies on behavioral intention have been conducted in developed countries [19, 20], a growing literature has focused on developing countries, such as Malaysia [21–23], Indonesia [24], India [25, 26], Taiwan [27], and Vietnam [28]. Mutual funds research has highlighted conventional funds in Malaysia [1, 21, 22] and India [29]. Only a few studies have examined IUT in Indonesia [30] and Malaysia [31]. Sumiati et al. [30] analyzed the influence of attitudes, subjective norms, and religiosity on the intention to invest among the millennial generation in Indonesia using the Theory of Reason Action (TRA). Yusuff et al. [31] investigated the effect of product knowledge and information sources on IUT investment decisions using the Engel, Blackwell, and Miniard (EBM) model.

However, previous literature has failed to examine the role of technology skills in influencing IUT investment behaviors. Fintech and IT innovations have made financial products and services becoming digital and more complex [32]. Plus, the Covid-19 pandemic has accelerated the cashless society culture and the use of digital platforms. Thus, investors need fintech self-efficacy (FSE) to make investment decisions. This study examined the influence of FSE on IUT investment intention. Specifically, it extended the Theory of Planned Behavior (TPB) model by replacing perceived behavioral control (PBC) with Fintech self-efficacy. Additionally, it added IFL and religiosity to the model. The TPB has been examined in various domains, such as halal transportation [6], internet purchasing [33], Takaful schemes [34], and Islamic banking depositors [35]. Similarly, in investment study, the TPB is dominant, given the significant effect of attitude, subjective norms, and PBC in influencing investment intention [24, 33].

The findings provide theoretical and practical implications. Theoretically, the study re-evaluates the framework of the TPB in the context of Shariah-compliant investment, focusing specifically on the IUT. It extends the TPB model by replacing Perceived Behavioral Control (PBC) with Fintech Self-Efficacy (FSE). Previous research on self-efficacy has mostly focused on domains such as computer self-efficacy in e-banking [36], self-efficacy in online health services [37], and financial self-efficacy in stock market investment [25] and financial inclusion [38]. However, in the context of IUT investment in Malaysia, replacing PBC with FSE is an appropriate and timely modification, in line with the increased digitization of the investment process, where the influence of Fintech is important. FSE, characterized as a measure of individuals’ confidence in their ability to use financial technology efficiently, provides a more relevant and contextually accurate view of this digital financial landscape than the broader scope of PBC. By specifically addressing the skills and confidence required for proficient use of online investment platforms, these adjustments significantly improve the predictive accuracy of the TPB model. Emphasizing FSE over PBC allows the model to more effectively reflect the technological competencies important to contemporary investors in Malaysia, making it a superior and more applicable tool in predicting investment intentions in a technologically advanced financial environment. Additionally, this study explored the effect of FSE as a moderating variable between attitude and intention. Testing the moderating role of FSE is important because it reveals how personal confidence in using Fintech tools can significantly amplify or reduce the effect of positive attitudes on investment actions. This approach introduces an important dimension to the behavioral finance framework, particularly in the growing Fintech and Islamic finance sectors in Malaysia, by showing that the effectiveness of attitudes in predicting investment intentions varies according to individual technological proficiency.

Moreover, two additional predictors were added to the TPB, i.e., IFL and religiosity. IFL, which includes knowledge and understanding of Islamic financial principles, provides an important cognitive foundation that shapes individuals’ attitudes and perceptions towards Islamic financial products, thereby directly influencing their investment intentions. Religiosity, on the other hand, reflects the extent to which Islamic values ​​and principles are embedded in an individual’s decision-making process, offering an additional layer of understanding about the motivations behind choosing Shariah-compliant investments. By integrating these factors into the TPB model, the analysis becomes more culturally and context-sensitive, offering deeper insights into the complex interplay of knowledge, religious beliefs and behavioral intentions in the field of Islamic finance in Malaysia. Also, it tested the role of attitude as a mediator between these two variables and intention to invest in IUT. Attitude, as a mediator, provides insight into how IFL and religiosity translate into actual investment decisions. Although IFL equips individuals with the knowledge and understanding of Islamic financial principles, and religiosity embeds this investment in a moral and ethical framework, it is the attitude resulting from these factors that realize the intention to invest. This mediation emphasizes the psychological process through which education (IFL) and personal beliefs (religion) are not only internalized but also transformed into actionable financial behavior. Examining attitude as a mediator in this context is particularly relevant in Malaysia, where the Islamic financial market is sophisticated and diverse, offering a rich environment for understanding how cognitive and cultural factors jointly influence investment choices in Islamic financial products.

From a practical perspective, these insights offer valuable guidance to policymakers and industry in understanding investor perceptions and behavior. For fund managers, understanding the determinants of investment intention in IUT enables the development of target strategies that not only increase investment in IUT but also shape marketing and communication approaches to align with the values and social context of potential investors. Policymakers can use these findings to design effective programs and infrastructure that foster positive attitudes toward IUT investment. Additionally, investors can benefit from these insights by making more informed investment decisions. In the context of Malaysia, where Islamic finance plays an important role in the economy, this understanding is important to stimulate growth and participation in the sector, thereby contributing to the development of the wider Islamic finance industry.

## Literature review

### Theoretical foundation

The study aims to examine consumer behavior determinants, specifically the intention to invest in the IUT context. In the TPB, intentions act as "the motivating factors that influence behavior," with intentions determining a person’s effort to perform a behavior [39]. We use TPB over the Technology Acceptance Model (TAM) or Unified Theory of Acceptance and Use of Technology (UTAUT) because it takes into account the role of intentions in determining behavior, which is considered an important mediator between attitudes and behavior. In addition, TPB also accounts for the influence of subjective norms on behavior, an important factor not captured by the other two models.

Fintech and investment cannot be detached. Fintech is an industry that continues to grow, replacing established financial services with innovative features, convenience, reach, and cost [40]. Fintech is revolutionizing the financial services industry at an unprecedented rate [11], from mobile payments, robo-advisor platforms, and application-based investments, to online banking solutions [12]. The development of fintech has affected financial planning, financial well-being, and economic inequality [41]. Leong [42] defines fintech as any innovative idea that improves financial service processes by proposing technological solutions according to business situations, and that idea can also lead to new business models or new businesses.

At the same time, advanced financial technology must be accompanied by the ability and confidence of users for it to function effectively and have the optimal impact. The investment process will be less efficient without fintech skills and self-efficacy. Self-Efficacy Theory (SET), as formulated by Bandura [43], posits that self-efficacy refers to an individual’s conviction regarding their capacity to effectively organize and execute certain actions in order to attain desired objectives. Self-efficacy is one of the most influential factors in the performance of a behavior because it can boost a person’s confidence [25, 44]. Moreover, in the context of the COVID-19 pandemic, it is essential to perceive the role of self-efficacy in influencing the investment behavior of individuals applying for fintech facilities. However, studies in the context of self-efficacy are scarce, specifically in the contexts of computer self-efficacy [36, 45, 46], health and mobile self-efficacy [37], and financial self-efficacy [25, 38].

Apart from the common TPB predictors (attitude, subjective norms, and PBC), pertinent constructs can be incorporated to improve predictability, which enhances the understanding of investment behavioral intention [47]. This study enriches investor behavior studies by extending the TPB model with IFL and religiosity, with attitude serving as a mediator and FSE as a moderator. Specifically, the TPB’s ability in predicting consumer behavior towards IUT was improved. The findings provide insights for Shariah-compliant mutual fund service providers, fund managers, and Muslim consumers worldwide.

### Behavioral intention

Behavioral intention is a person’s determination to place effort into attempting a reality [39]. Intention represents a person’s motivation, plan, or earnest decision to exert efforts and subsequently behavior. Fishbein and Ajzen [48] suggest that intentions are the most reliable indicator of actual behavior, where the likelihood that the behavior will be performed increases with the strength of the intentions. Several studies have measured the influence of certain factors on the intention and behavior of users in various Islamic finance industries, such as Islamic insurance (takaful) [34, 49], banking [19, 50], and bonds (sukuk) [51, 52]. This study examined the factors determining the intention to invest in IUT.

### Attitude

Attitude denotes a person’s positive or negative judgment of a specific behavior [53]. Allport [54] describes attitude as a mental state that can directly or indirectly impact a person’s response to all the connected things and events. Numerous studies have demonstrated attitude as one of the most significant influencers on consumer behavior. Raut [55] discovered a positive relationship between attitude and intention to invest in the Indian stock market. Other studies highlighted a positive relationship between attitude and intention to use Islamic credit cards [56] and the adoption of Islamic banking [35]. Thus, this study proposed the following hypothesis:

H1: Attitude is positively related to IUT investment intention.

### Subjective norms

The second factor influencing behavioral intention is subjective norms, which reflect the individual’s perception of social influence and the expectations of their social environment, including friends and family [57]. Subjective norms are important in shaping investment decisions, acting as a form of social pressure or encouragement based on the approval or disapproval of influential people in a person’s life. Research has demonstrated its varying effects. Several studies have emphasized the importance of subjective norms in influencing investment intentions in IUTs in Malaysia [58, 59], deposits in Islamic banking in Malaysia [35], Islamic credit cards in Pakistan [56], and socially responsible investments in India [60]. However, others suggest that more knowledgeable investors may rely less on these social norms and more on their personal expertise, especially in IUTs [58, 61, 62]. The significance of this variable in the current Malaysian society is an interesting study topic; hence the following hypothesis was proposed:

H2: Subjective norms are positively related to IUT investment intention.

### Fintech self-efficacy

Self-efficacy is an individual’s belief in their ability to successfully perform the behaviors required to produce a desired outcome [38]. Self-efficacy is a dynamic property that emerges in various contexts, which can be changed by certain individual behaviors, biological events, and their environment [44]. Unlike PBC, which includes a broader perception of control over behavior, self-efficacy focuses more on a person’s confidence in their personal abilities such as skills and competence to perform a specific task, reflecting their belief as to whether they can effectively perform the actions required to achieve a specific goal. Self-efficacy provides a more targeted measure of individuals’ confidence in their ability to perform a behavior, thereby offering a more direct link to their behavioral intentions and actions in a specific context. For example, past studies examined the influence of financial self-efficacy on stock market investment in India [25] and financial services in Uganda [38]. Considering technology development, more studies have modified self-efficacy by investigating the role of computer self-efficacy. The significant effect of computer self-efficacy has been evidenced in the context of e-banking in Jordan [36], computer facilities in England [46] and mobile banking in India [63]. In this study, Fintech self-efficacy (FSE) is adopted to measure individuals’ confidence in their ability to efficiently use Fintech by addressing the skills and confidence required for proficient use of online investment platforms [64]. This is in line with [41, 65], emphasizing the significant influence of Fintech development in changing financial planning and well-being. Therefore, it is important to understand the role of self-efficacy in influencing individual behavior to invest through fintech facilities. Hence, this study suggested the third hypothesis as follows:

H3: FSE is positively related to IUT investment intention.

### Islamic financial literacy

Financial literacy, according to Atkinson and Messy [66], is a combination of awareness, knowledge, skills, attitudes, and behaviors that can improve the quality of financial decisions and achieve individual financial well-being. Meanwhile, IFL is an individual’s ability, skills, and attitude to understand and analyze financial information based on Islamic perspectives [67]. Antara et al. [68] defined IFL as the level of individual knowledge, awareness, and skills to understand the basis of information and Islamic financial services, influencing their attitude toward making appropriate Islamic financing decisions. Previous studies have highlighted the impact of IFL on people’s intention to use Islamic banking in the UAE [19, 69] and Oman [70]. Meanwhile, financial literacy has influenced financial decision-making in the United Kingdom and Malaysia [71], United States [72], Belgium [73], and Cyprus [74].

Financial literacy significantly affects individuals’ financial attitudes by improving their understanding of risk and return, thus increasing confidence in making informed financial decisions. For example, financial literacy enabled SMEs to form a positive risk attitude toward investing in profitable business opportunities in Sri Lanka [75]. A positive association between financial literacy-risk attitude is also observed in Taiwanese derivative market participation [76]. Having literacy in finance fosters a deeper appreciation of the importance of diversification and long-term planning [77]. This comprehensive understanding and awareness leads to a more calculated, less emotionally driven investment approach, often characterized by a strategic, goal-oriented perspective rather than a speculative or risk-averse stance. Financially literate individuals are better at navigating market conditions, seeing relevant information from the vast amount of data available, and reducing common behavioral tendencies such as overconfidence or herd mentality. This argument aligns with [55], evidencing the positive financial literacy-investment attitude in the Indian stock market.

Alshater et al. [78] suggested that future research should investigate whether financial literacy and religiosity influence preference for Islamic finance over conventional finance. Islamic financial literacy (IFL) greatly influences investors’ attitudes towards Shariah-compliant investments by applying an understanding of key Islamic financial principles, such as the prohibition of interest (Riba) and ethical investment norms. Investors who are knowledgeable in this area tend to choose investments that comply with Islamic law, showing a higher tendency for risk-averse and ethical choices. This literacy encourages preference for certain financial products such as IUT and shapes a more holistic investment approach that balances financial returns with religious, ethical and social considerations. Therefore, IFL not only guides financial decisions but also aligns them with broader ethical and spiritual values, leading to distinctive investment patterns in the field of Sharia-compliant finance. The study proposed the following hypotheses based on the aforementioned discussion:

H4: IFL is positively related to IUT investment intention.
H5: IFL is positively related to attitude.

### Religiosity

Religiosity or religious commitment is the degree to which a person adheres to religious values, beliefs, and practices and applies them in daily life [79]. Religion affects human behavior and attitudes [80, 81]. Religiosity plays a dominant and influential role in Muslim consumers’ attitudes in Bangladesh [82] and UAE [83]. Duqi and al-Tamimi [16] state that religiosity is a prominent predictor of UAE investors’ investment in sukuk. In Jamaludin and Gerrans [84], religiosity is closely related to an individual’s decision in choosing the type of fund. The analysis of EPF investors revealed that casual and liberal Muslims prefer conventional funds, while obedient and devoted Muslims favor Islamic funds, which suggests that religion influences an individual’s mutual fund investment decision. Hence, this study proposed the following:

H6: Religiosity is positively related to attitude.
H7: Religiosity is positively related to IUT investment intention.

### Mediation

This study examined the mediating role of attitude in investigating IUT investment intention, which develops the inference quality and substantially contributes to the study [85]. Previous studies suggested the vital role of attitude as a mediator of behavioral intention in financial services, specifically between religiosity and purchase intention towards Islamic insurance in Bangladesh [82], financial knowledge and investment intention in India [25], and halal brand purchasing between religiosity and halal brand purchase intention in India [86]. In Islamic banking adoption, Albaity and Rahman [19] suggested attitude as a fully mediating factor for customers’ behavioral intentions between IFL and intention to use Islamic banking in UAE. Souiden and Rani [87] discovered that attitude mediates religiosity and the purchase intention of Islamic bank services in Tunisia. Therefore, attitude mediates the relationship between IFL and investment intention and the relationship between religiosity and investment intention:

H8: Attitude mediates the relationship between IFL and investment intention.
H9: Attitude mediates the relationship between religiosity and investment intention.

### Moderation

A moderator can increase the ability to predict outcomes in a study. Moderating factor affects the strength or direction of the relationship between an independent or predictor variable and a dependent or criterion variable [85]. Moderating variables are often used in unreliable or contradictory studies. Although most studies discovered that behavioral intention and TPB characteristics are consistently positively correlated, some have revealed the opposite [88, 89]. Thus, incorporating a moderator in testing the attitude-intention relationship is valid. Previous studies found a positive moderating effect of self-efficacy on the relationship between attitude and entrepreneurial intention in Vietnam [90]. The significant moderating role is also supported the earlier findings by [45] who found that computer self-efficacy strengthens the relationship between attitude and intention to switch to online banking in Taiwan. Other studies found that financial self-efficacy positively moderates attitude-stock market participation relationship in India [25] and Pakistan [91].

This study scrutinizes the impact of attitude on IUT investment intention, with a focus on FSE as a moderating factor. FSE plays an important role as a moderator as it could influence investment intention by either strengthening or weakening the intention to invest. Even with a positive attitude, low confidence in using fintech tools can reduce the intention to invest. On the other hand, high FSE can increase investment intention, empowering individuals to invest even if their attitudes are not very positive. It can also overcome negative attitudes by instilling the confidence to take calculated risks and explore new investment opportunities. Therefore, improving FSE is as important as fostering a positive investment attitude. The FSE could be a significant moderator of the IUT investment intention. Thus, the following hypothesis was presented:

H10: The positive relationship between attitude and investment intention will be stronger when FSE is high.

**Fig 1:**
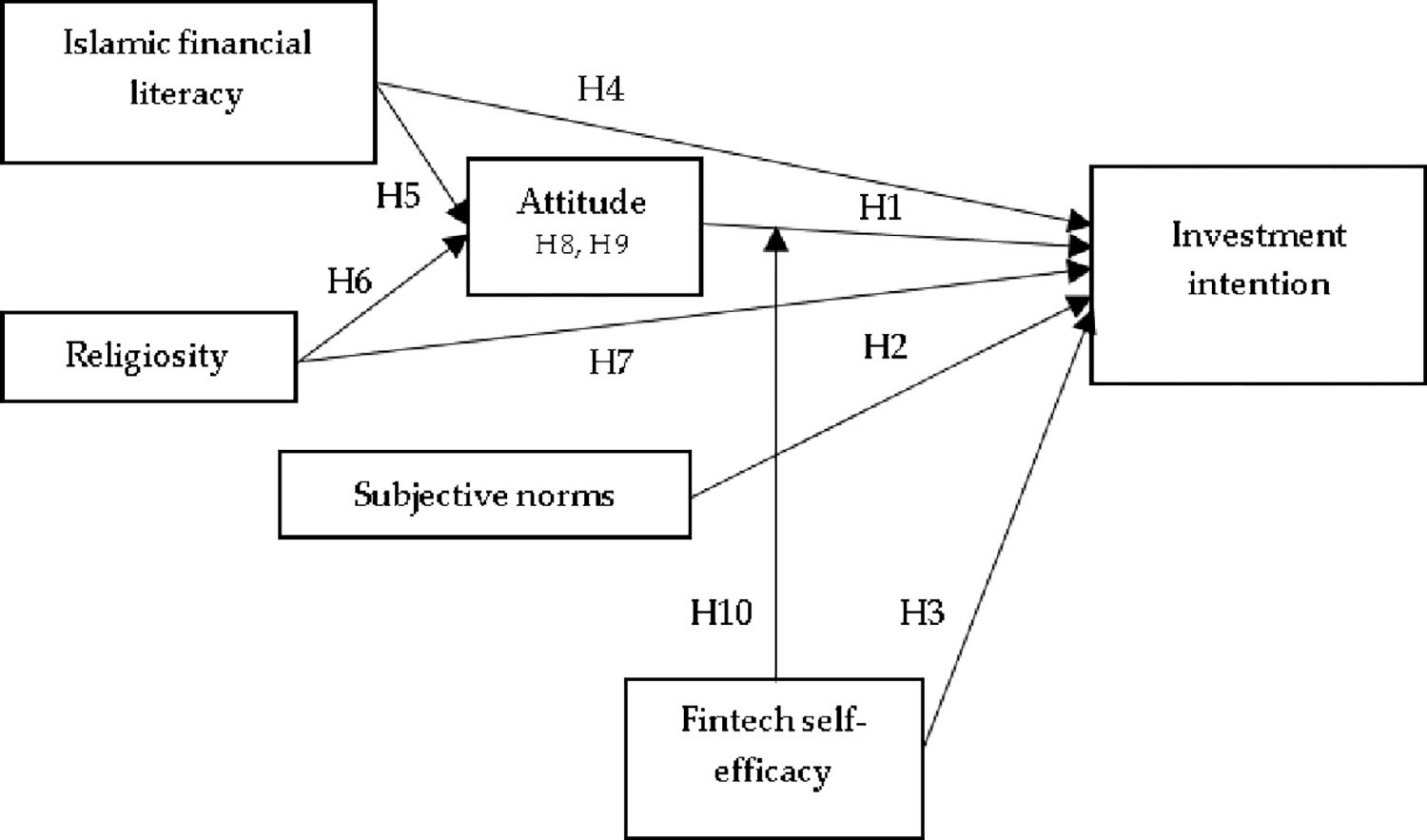
Research framework.

Fig 1 displays the research model and includes all the proposed study relationships.

## Research methodology

### Research design

This study used a quantitative research methodology in the form of surveys to obtain standard and comprehensive information to determine the association between constructs. In addition, this study was also categorized as a cross-sectional study. A structural equation modeling using Smart Partial Least Squares (SmartPLS) 4.0 was used to test the proposed hypotheses. Sampling techniques, study instruments, and ethics statement were explained in the following sub-sections.

#### Sampling technique and data collection

The study focused on Muslim investors with fintech experience and used a purposive sampling method, where participants were purposively selected to examine Muslim investors’ intention to invest in IUT. To effectively reach this specific group, snowball sampling was used. It starts with a small group of participants who meet the criteria by approaching the leading fund management firms, including Public Mutual Berhad, Kenanga Investors Berhad, PMB Investment Berhad, and RHB Islamic International Asset Management Berhad. They assisted in distributing online questionnaires to their clients, and asked them to refer other potential participants. This process continues until the desired number of respondents is reached. We adopted a snowball sampling approach due to the unavailability of participant lists and to reduce the potential for refusal to participate, with no reward offered to respondents.

The respondents for the sampling frame were selected based on three screening questions to ensure they matched the aforementioned sampling criteria. Firstly, the respondents must be Muslims over 18 years old. The second inquiry concerned past fintech service usage and the specific service used. Thirdly, the respondents were asked about their history in IUT investment. A total of 417 data were collected, but 25 were eliminated following the straight-lining and outliers review procedure. Thus, the final data amounted to 392. The sample size is considered acceptable as the minimum sample size required based on the G-power analysis is only 138.

#### Scale and measurement

The measured constructs were adapted from and validated based on previous studies (see S1 Appendix). The five items used to measure investment intention were adapted from Lai [27]. Meanwhile, attitude was measured based on four items adapted from Akhtar and Das [25] and Shahab Aziz et al. [49]. Subjective norms was evaluated using four items adapted from Amin [92]. The FSE construct was assessed based on five items modified from Mindra and Moya [38] and Lee et al. [45], while the six items used in evaluating IFL were adapted from Abdul Rahim et al. [67]. Lastly, religiosity was measured using ten items from Mahdzan et al. [22].

All exogenous variables were measured with a five-point Likert scale, a more suitable method for the general population who are less familiar with questionnaires [93]. Additionally, the endogenous variables (investment intention) were measured with a seven-point scale. Employing different Likert scales reduces the common method variance (CMV) [94] with an added marker variable. An expert validation was conducted before distributing the questionnaire survey, where five academic and business experts reviewed the instrument. The study ensured that the instrument could be clearly understood before data collection by conducting a pre-test cognitive interview with 15 community members. Subsequently, a pilot test was conducted among 30 respondents with similar characteristics within the target population. The actual questionnaire distribution was conducted in May and June 2022.

#### Ethics statement

The author received ethical approval for the study from the Research Ethical Committee at the National University of Malaysia, ensuring that the study complied with all applicable ethical standards. Data collection occurred between June 13, 2023 and July 10, 2023. Accompanying the questionnaire, an introduction letter outlined the purpose of the study and requested written consent from the participants. Before beginning their participation and submitting their responses, respondents were instructed to carefully review the ethical statement. They were assured that their information would be used solely for research purposes and would remain confidential.

## Data analysis

We employed the Partial Least Squares Structural Equation Modeling (PLS-SEM), specifically using SmartPLS 4.0, to address the exploratory nature and non-normality issues in this study [95]. The PLS-SEM facilitates the examination of complex variable relationships without the constraints of strict data distribution assumptions. Data analysis and reporting involved two stages; the first stage tested the measurement model construct validity and reliability, while the second stage analyzed the structural model support for the conceptual model [96]. The complexity of the research model, which includes two mediating hypotheses, one moderating hypothesis, and six direct hypotheses, highlights the suitability of PLS-SEM for our study [97–99]. The PLS-SEM is also suitable for studies with limited sample sizes.

### Common method variance

The CMV tests are essential in data collected via self-administered questionnaires, specifically when the same person provides the predictor and criterion variables [94]. This study used five-point and seven-point Likert-type scales for all independent and dependent variables, respectively, based on Podsakoff et al.’s suggestion.

Kock’s [100] full collinearity analysis was applied to establish that CMV was not severe in the current study. Therefore, CMV is established if the variance inflated factor (VIF) exceeds or is equal to 3.3. Given that all VIF values were below the recommended threshold value, the CMV was not severe.

A marker variable was assessed for CMV, which entailed using a source measure incorporating method variance as a covariate in the statistical analysis [94]. Using Ronkko and Ylitalo’s [101] method, the marker variables for this study were derived from Lin et al. [102], which consists of three unrelated items. The addition of marker variables did not significantly change the Beta (β) value or the path coefficient and R^2^, demonstrating that the data is free of CMV issues.

### Multivariate normality

Kline [103] stated that the normal multivariate skewness value is ≤ 3 and the normal kurtosis value is ≤ 20. Multivariate normality in this study was assessed using the Web Power online tool, which evaluates Mardia’s multivariate skewness, kurtosis coefficients, and p-values. Resultantly, the pooled data skewness (β = 9.941, p < 0.01) and kurtosis (β = 93.401, p < 0.01) were not multivariate normal. Thus, the results align with the SmartPLS requirements as a non-parametric software data analysis.

### Profile of respondents

The questionnaire was distributed using a Google form and 410 valid responses were received. The 18 invalid responses were identified during the straight-lining process. Of 392 respondents, 52.6% were female, and 41.9% were between the ages of 41 and 50. Most respondents from the public sector (53.1%), have more than 21 years of work experience (25.8%), hold bachelor’s degrees (45.2%), and have a monthly income from RM4000 to RM8000 (45.1%). Table 1 provides a summary of the respondent profile for this study.

**Table 1:** Profile of respondents.

	Category	N	%
Gender	Male	186	47.4
	Female	206	52.6
Age group	18–30 years	57	14.5
	31–40 years	115	29.3
	41–50 years	164	41.8
	51–60 years	39	9.9
	61 years and above	17	4.3
Education	Secondary school	18	4.6
	Certificate/Diploma	70	17.9
	Bachelor’s Degree	177	45.2
	Master’s Degree	88	22.4
	PhD	39	9.9
Employment	Public Service	208	53.1
	Private Service	92	23.5
	Business/Self-employed	37	9.4
	Student	28	7.1
	Pensioner	19	4.8
	Not working	8	2.0
Monthly income	Less than RM2000	24	6.1
	RM2001-RM4000	53	13.5
	RM4001-RM6000	91	23.2
	RM6001-RM8000	86	21.9
	RM8001-RM10000	42	10.7
	RM10001 and above	68	17.3
	No fixed income	28	7.1
Working experience	Less than five years	38	9.7
	5–10 years	48	12.2
	11–15 years	87	22.2
	16–20 years	83	21.2
	Over 21 years	101	25.8
	N/A	35	8.9

### Measurement model assessment

**Fig 2:**
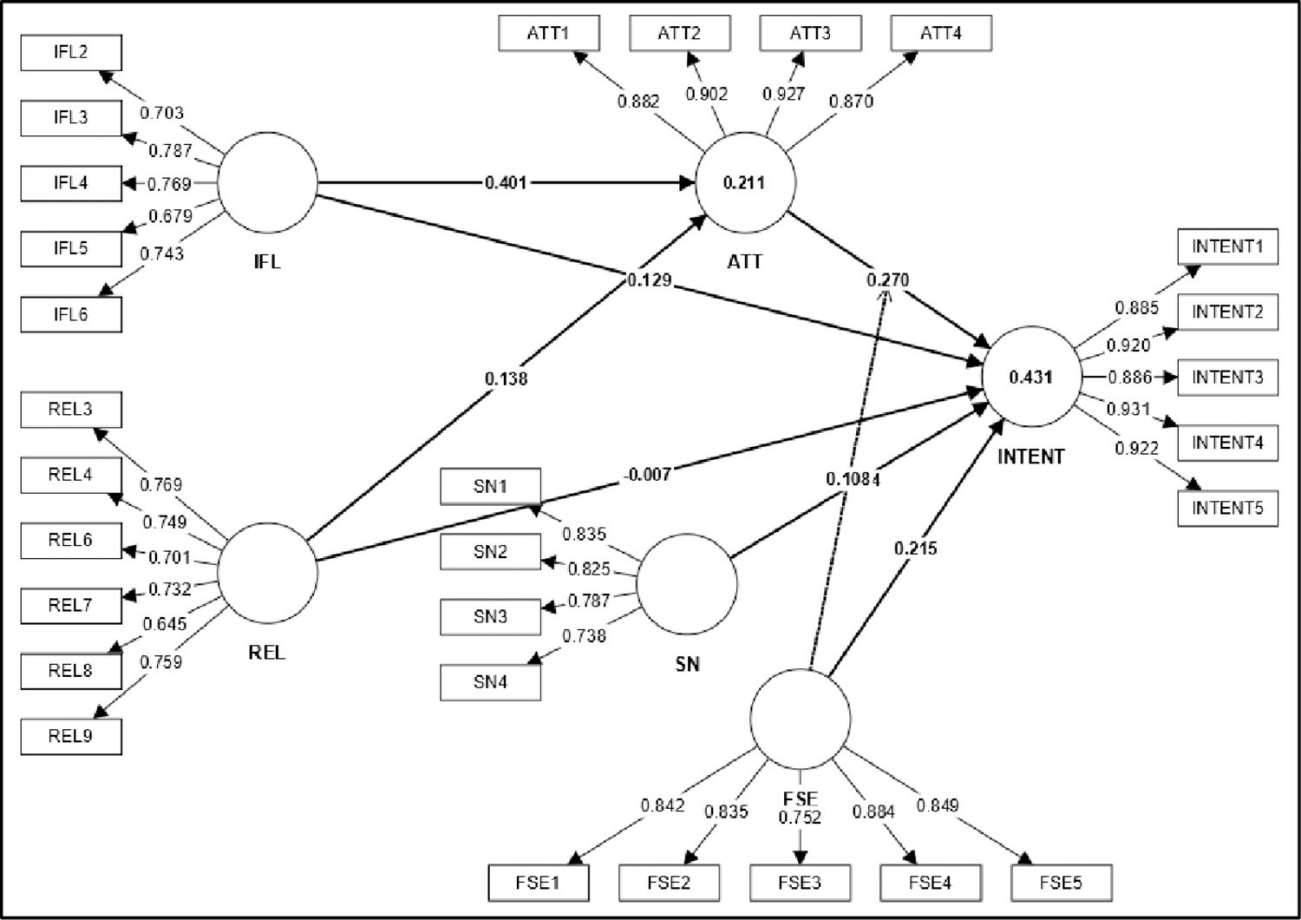
Measurement model assessment.

The consistency and reliability of the measurement model were assessed using Cronbach’s alpha and the composite reliability index. Cronbach’s alpha ranges from 0.790 to 0.947, where an acceptable range is a value of 0.7 or above [99]. In this study, CR ranged from 0.856 to 0.960, and AVE ranged from 0.529 to 0.826, which meets the minimum requirements [99]. Therefore, the convergent measurement was valid and reliable (refer to Fig 2 and Table 2).

**Table 2:** Indicator reliability, internal consistency reliability, and convergent validity.

Variable	Item	Indicator	Internal Consistency		Convergent
		Reliability	Reliability		Validity
		Outer Loadings	Composite Reliability (CR)	Cronbach’s Alpha	Average Variance Extracted (AVE)
		>0.70	>0.7	>0.7	>0.50
**Islamic financial literacy (IFL)**	IFL2	0.703	0.856	0.790	0.544
	IFL3	0.787			
	IFL4	0.769			
	IFL5	0.679			
	IFL6	0.743			
**Religiosity (REL)**	REL3	0.769	0.870	0.822	0.529
	REL4	0.749			
	REL6	0.701			
	REL7	0.732			
	REL8	0.645			
	REL9	0.759			
**Fintech self-efficacy (FSE)**	FSE1	0.842	0.919	0.889	0.695
	FSE2	0.835			
	FSE3	0.752			
	FSE4	0.884			
	FSE5	0.849			
**Attitude (ATT)**	ATT1	0.882	0.942	0.918	0.802
	ATT2	0.902			
	ATT3	0.927			
	ATT4	0.870			
**Subjective norms (SN)**	SN1	0.835	0.874	0.808	0.636
	SN2	0.825			
	SN3	0.787			
	SN4	0.738			
**Investment intention (INTENT)**	INTENT1	0.885	0.960	0.947	0.826
	INTENT2	0.920			
	INTENT3	0.886			
	INTENT4	0.931			
	INTENT5	0.922			

Note: IFL1, REL1, REL2, REL5, and REL10 were deleted due to low loadings

### Discriminant validity

Discriminant validity was established using the heterotrait-monotrait ratio (HTMT) [104]. Table 3 indicates that the HTMT values for all the constructs were under HTMT.85. Thus, discriminant validity was established in this study.

**Table 3:** HTMT criterion.

	IFL	REL	FSE	ATT	SN	INTENT
**IFL**						
**REL**	0.349					
**FSE**	0.434	0.202				
**ATT**	0.505	0.279	0.267			
**SN**	0.513	0.314	0.250	0.672		
**INTENT**	0.477	0.234	0.404	0.553	0.617	

### Structural model assessment

**Fig 3:**
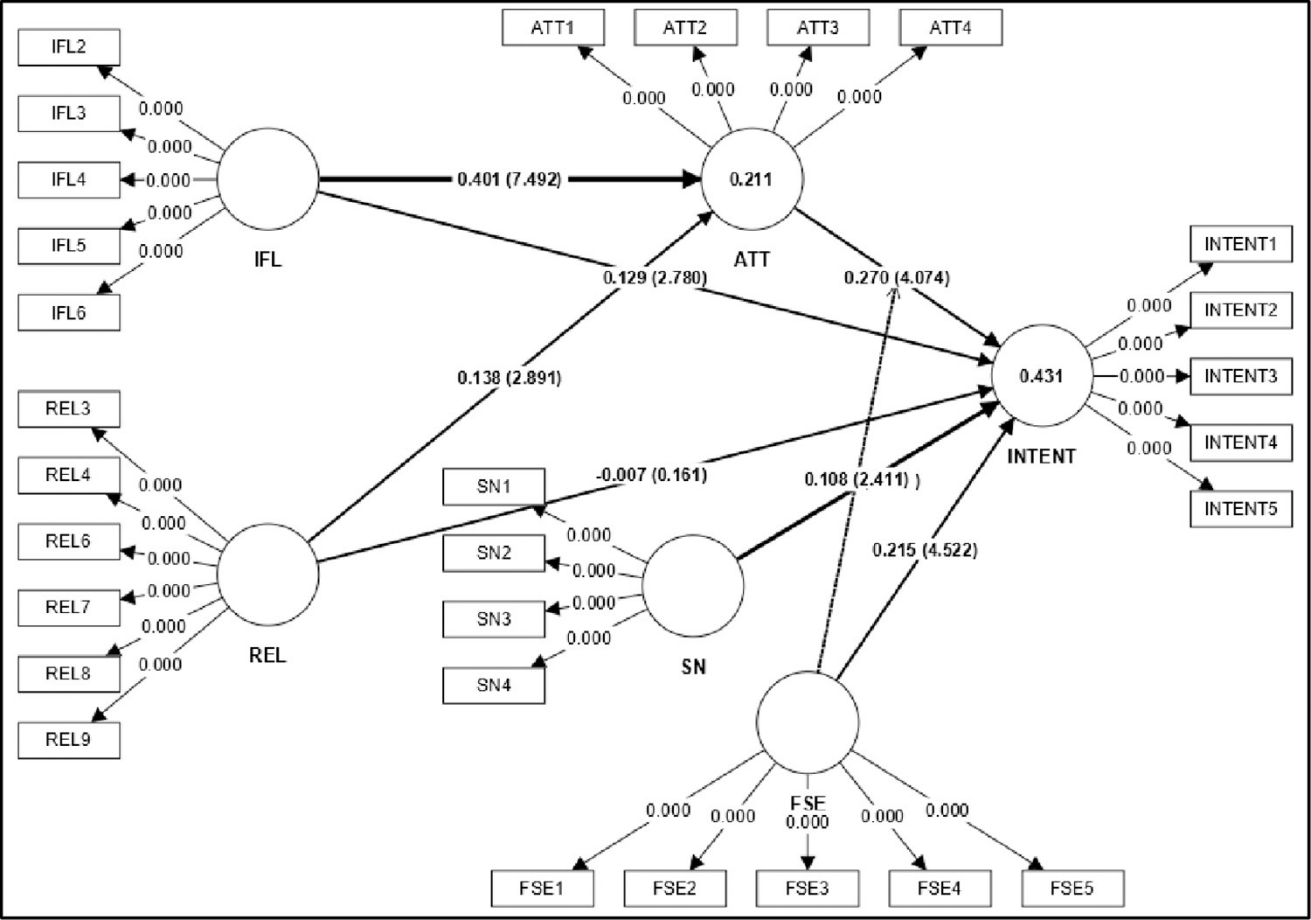
Structural model assessment.

Fig 3 and Table 4 illustrate the association among several variables of the conceptual model and highlight the hypothesis criteria developed in the study. Only one of the nine direct hypotheses was unsupported. The relationship between IFL and INTENT was significantly positive (β = 0.129, p < 0.01), ATT (β = 0.270, p < 0.01), SN (β = 0.304, p < 0.01), and FSE (β = 0.215, p < 0.01). Surprisingly, the relationship between REL and INTENT was not supported (β = -0.007, p = 0.490). Thus, H1, H3, H4, and H5 were supported, while H2 was not supported. The relationship between IFL and REL with ATT was also significantly positive (β = 0.401, p < 0.001) and (β = 0.138, p < 0.01), respectively.

**Table 4:** Hypothesis testing result.

		Std. Beta	Std. Dev	t-value	p-value	BCI LL	BCI UL	Decision	f^2^	R^2^	Q^2^
**H1**	ATT -> INTENT	0.270	0.066	4.074	0.000	0.162	0.378	supported	0.060	0.431	0.350
**H2**	SN -> INTENT	0.304	0.062	4.918	0.000	0.203	0.403	supported	0.105		
**H3**	FSE -> INTENT	0.215	0.048	4.522	0.000	0.137	0.293	supported	0.064		
**H4**	IFL -> INTENT	0.129	0.047	2.780	0.003	0.053	0.206	supported	0.013		
**H5**	IFL -> ATT	0.401	0.054	7.492	0.000	0.307	0.484	supported	0.188	0.211	0.196
**H6**	REL -> ATT	0.138	0.048	2.891	0.002	0.054	0.211	supported	0.022		
**H7**	REL -> INTENT	-0.007	0.043	0.161	0.436	-0.080	0.060	not supported	0.000		
**H8**	IFL -> ATT -> INTENT	0.096	0.029	3.272	0.001	0.053	0.151	supported			
**H9**	REL -> ATT -> INTENT	0.033	0.015	2.269	0.012	0.014	0.062	supported			
**H10**	ATT*FSE -> INTENT	0.108	0.045	2.411	0.008	0.036	0.185	supported			

These factors explained 43.1% (R^2^ = 0.4316) of the variance in investment intention and 21.1% (R^2^ = 0.211) of the variance in attitude. In Table 4, IFL did not affect (f^2^ = 0.013) on INTENT, while ATT (f^2^ = 0.060) and FSE (f^2^ = 0.064) demonstrated a small effect on INTENT. Meanwhile, SN had a moderate effect on INTENT (f^2^ = 0.105). Moreover, no 0 straddles were observed between the Lower and Upper Limits [104], excluding REL-INTENT. Therefore, all results were significant except for the religiosity-investment intention relationship. The Q^2^ value of 0.35 (exceeded zero), which signifies the high predictive power of the model. implies the sizable predictive significance of the model [99].

**Fig 4:**
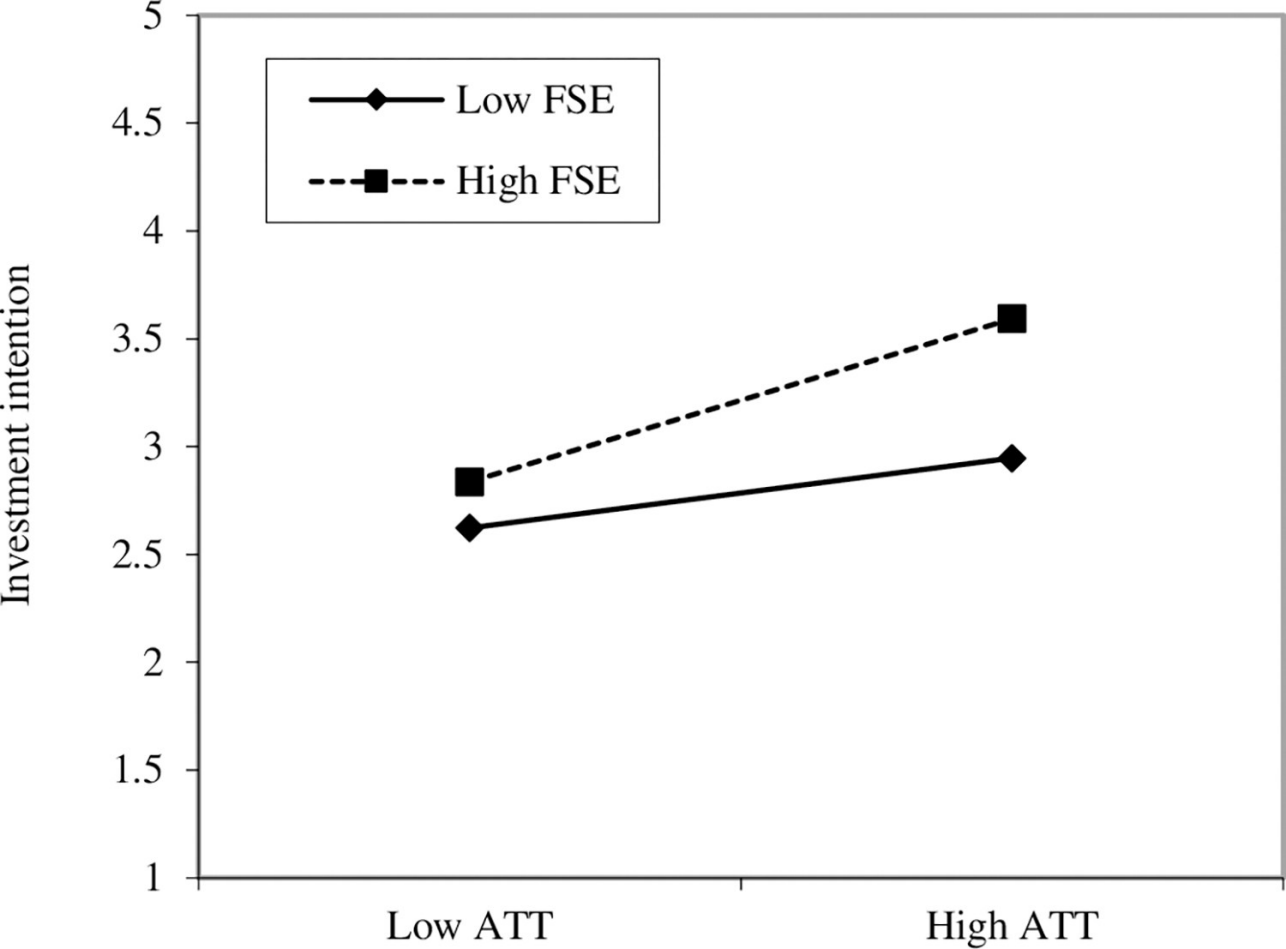
Interaction plot.

Preacher and Hayes [105] guidelines were applied to test the mediation analysis by bootstrapping the indirect effect. Resultantly, the relationship between IFL -> ATT -> INTENT (β = 0.096, p < 0.01) and REL -> ATT -> INTENT (β = 0.033 p < 0.01). Hence, attitude mediates both relationships, thus supporting H8 and H9. The moderation effect of FSE on the relationship between ATT and INTENT indicated a significantly positive interaction (t-value = 2.411; p-value = 0.008). Fig 4 illustrates a simple slope analysis of the moderating effect of FSE on attitude and investment intention. The interaction plot denotes that the relationship between ATT and INTENT is stronger when the FSE is high, thus supporting H10.

### Predictive assessment

Shmueli et al. [106] stated that PLSpredict is a sample-based procedure that generates predictions at the item or constructs level with a 10-fold procedure to determine predictor relevance. Additionally, a comparison should be performed on the differences in PLS and item values in the linear regression model (LM). The PLS-SEM with lower values than LM indicates higher predictive power. Table 5 demonstrates the PLSpredict results where all errors from the PLS model are lower than the LM, which suggests strong predictive power. The Q^2^ values exceeded zero, which signifies the high predictive power of the model.

**Table 5:** Predictive model assessment.

	PLS-SEM_RMSE	LM_RMSE	PLS-LM RMSE
INTENT1** **	0.935	0.973	-0.038
INTENT2** **	0.908	0.938	-0.030
INTENT3** **	1.088	1.117	-0.029
INTENT4** **	0.979	1.009	-0.030
INTENT5** **	1.040	1.074	-0.034

Note: PLS = partial least squares; RMSE = root mean squared error; LM = linear regression model

## Discussion

This study analyzes the factors influencing individuals’ intentions to invest in IUT in Malaysia. A research model based on the TPB was tested to gain insights into the factors. This model also replaces the PBC in the TPB with the FSE to determine if this factor can influence the intention. There are ten examined hypotheses, and the results of the study indicate that nine hypotheses are supported, and just one is not supported. All TPB dependent variables, namely attitude, subjective norms, and FSE, have a positive and significant relationship with IUT investment intention. These findings are consistent with the TPB’s theoretical framework, which assumes that the individual context (attitude and PBC) and the environment (subjective norms) are driving variables in the adoption of a behavior.

Subjective norms have a positive relationship with IUT intention with the largest magnitude, followed by attitude, FSE, and IFL. Hence, societal pressure influences individual behavior in Malaysia which confirms previous findings in other Islamic finance areas. For instance, subjective norms are significant in Islamic credit cards in Pakistan [56] and Islamic banking in Malaysia [35]. The findings regarding conventional investment support Lai’s [27] work in the Taiwan stock market. Influential individuals’ (family and friends) opinions is crucial in IUT investment intention. Consistent with behavioral finance studies, an individual’s decision-making is influenced by psychological elements, such as herding behavior [23]. Investors make decisions based on the cognitive bias of their social circles, such as friends and family. The results also confirmed studies of high-context cultures, such as among the Chinese and Arabs [61,107], Japanese [108], and Malaysians [109]. Correspondingly, Malaysians are collectivist and prefer to comply with societal norms.

In addition, attitude was also found to have a significant relationship with investment intention. Attitude is a significant predictor of the behavioral intention to purchase Islamic banking products and services in Malaysia [110] and Pakistan [61]. Attitude is also crucial in sukuk investment intention among Libyan investors [107]. Therefore, Muslim customers and investors believe that Shariah-compliant products are essential in preserving Islamic principles of their wealth creation, which influences their attitudes. They also believe investing in IUT is a practical and wise choice and favor the idea of IUT investment. The results aligned with TPB, which states that attitude describes the extent to which a person has a positive or negative evaluation or judgment of the intended behavior [39].

A significant positive relationship was also revealed between FSE and IUT investment intention. Respondents are more confident in making investment decisions when they are technologically savvy and own the devices and infrastructures to conduct online investments. These findings aligned with past studies on the financial self-efficacy for the stock market [25] and financial inclusion [38], as well as computer self-efficacy for e-banking services [36]. Hence, technological skills need to be enhanced to motivate people to invest in IUT. Facilities and infrastructures are critical in facilitating investors. The presence of many investors results in a deeper market, which attracts more investors and boosts national financial development. This situation distinguishes the financial development between developed and emerging economies [111].

The findings disclosed that IFL has a significant positive relationship with attitude and IUT investment intention. Warsame and Ireri [69] highlighted similar results in the adoption of Islamic banking services among consumers in the UAE and Al-Balushi et al. [70] in small and medium enterprises (SMEs) in Oman. Financial literacy influenced financial decision-making in the United States [72] and Belgium [73], which significantly impacted stock market investors’ attitudes in India [55]. This study is primarily based on current financial literacy research, specifically concerning the characteristics of IFL that encourage investors’ IUT preference over conventional unit trust funds. The instruments in previous studies are based on conventional perspectives without explicitly outlining Islamic perspectives.

Therefore, IFL in this study used the same concept with an Islamic perspective, which is defined as "the capacity to apply financial knowledge, skill, and attitude in managing financial resources in accordance with Islamic teachings" [67]. This study investigated the relationship between subjective IFL and IUT investment intention. The IFL has been deeply engrained in Malaysian culture due to national regulations and being a Muslim-dominated country. Therefore, IFL significantly impacts Malaysian investors’ financial decisions, which explains the importance of IFL on investment intention for other Islamic financial products. The benefits of Islamic financial instruments, such as ethical investment, no involvement in speculative activities, and support from underlying assets, encouraged investment in Shariah-compliant products. Muslims who gain IFL tend to invest in Islamic financial products. Additionally, Islam demands that money be accumulated based on Shariah principles. Wise people will attempt to accumulate wealth in line with Shariah.

Religiosity influences attitude, but it is not significant towards IUT investment intention. The findings suggest the different ways that religious beliefs manifest in making financial decisions. Religion significantly shapes attitudes towards Islamic trust units, especially because these products are in line with Islamic principles, such as the prohibition of interest and ethical investment. Individuals who value these religious principles are likely to show positive attitudes toward such investments, as they resonate with their personal and religious ethos. However, when it comes to the actual intention to invest, several other factors play a role, reducing the direct influence of religion. For instance, lack of knowledge and awareness of IUT products could hinder their intention to invest. Moreover, financial considerations (i.e. return on investment, market dynamics and risk tolerance), psychological impediments (i.e. fear of uncertainties, resistance to change, or a lack of confidence), social influence could also influence of investment intention. In short, although religious beliefs shape basic attitudes, multifaceted and pragmatic aspects of investment decision-making often override these beliefs, highlighting the complexity and diversity of factors influencing financial choices. The insignificant religion-IUT intention relationship is consistent with earlier studies on portfolio allocation [112], intention to purchase halal brands [86], and willingness to pay for halal transportation [6]. Furthermore, [87] argued that certain Muslims may not adhere to spiritual principles when conducting financial and business transactions. Nonetheless, the finding contradicts previous studies on Islamic bonds (sukuk) in the UAE [16], ICM products (interest-bearing sukuk and stocks) in Bangladesh [82], and Islamic banking products in Kashmir [62].

Interestingly, although religiosity has no direct effect on investment intention, it has an indirect effect when mediated through attitude. Initially, religious beliefs, especially those related to Islam, influence individual attitudes toward IUT investment [6]. Muslim finds that IUT products, which adhere to Shariah principles, are in line with their religious values. This alignment fosters a positive attitude towards IUT, as it is seen as not only financially sound, but also morally and religiously congruent. The positive attitude then plays an important role in shaping their intention, thus more likely to consider the IUT as a viable investment option. It acts as a bridge between abstract religious beliefs and concrete financial actions. Thus, attitudes function as an important mediator, translating religious beliefs into actual investment intentions. It highlights the psychological pathways through which religious beliefs shape perceptions and evaluations, ultimately guiding investment behavior and decisions, particularly in contexts where financial choices are closely linked to ethical and religious considerations.

Attitudes also significantly mediated the relationship between IFL and investment intention, which confirms previous studies [19, 25, 75]. Improved Islamic financial literacy, which includes an understanding of key Islamic financial principles such as profit sharing and the prohibition of interest (riba), leads to the formation of positive attitudes towards IUT investment. This informed perspective recognizes the alignment of IUT with financial viability and religious principles. In turn, such a positive attitude, rooted in a comprehensive understanding of Islamic finance, significantly influences investment decisions. It acts as a vital link (mediator), converting Islamic financial knowledge and awareness into IUT investment intent.

The FSE moderated the link between attitude and investment intention. The influence of FSE strengthens the marginal impact of attitudes towards intention. This finding parallels Doanh [90] in Vietnam, where self-efficacy positively moderated the relationship between attitude and entrepreneurial intention. Similarly, Lee et al. [45] mentioned that computer self-efficacy positively moderated the attitude-online banking relationship among Taiwanese bank customers. The availability of skills and facilities strengthens the relationship between attitude and intention to invest in IUT. As banks, stock trading firms, and financial institutions have recently moved online [113], investment platforms, especially mutual funds, should move in a similar direction.

## Theoretical and practical implications

This study has some valuable contributions to the field of behavioral finance. Within the theoretical aspects, it contributes in four-fold. First, this study replaced PBC in TPB with FSE.

Replacing PBC with FSE significantly increases its applicability in the context of Fintech, particularly in examining investment intentions in Islamic trust units in Malaysia. FSE, a more specific construct, focuses on individuals’ beliefs in their ability to effectively use financial technology, thus providing a more accurate measure of behavioral control related to Fintech investment. This replacement not only improves the predictive power of the TPB model for Fintech-related intentions by directly addressing the required skills and competencies but also aligns the theory more closely with the unique Malaysian cultural and economic context, where Islamic finance and Fintech are prominent. This theoretical adjustment thus offers a more contextually appropriate and practically relevant tool for understanding and predicting investment behavior in the evolving digital financial landscape.

Second, in addition to the direct FSE-intention relationship, this study also examined the moderating role (indirect effect) of FSE in attitude-intention relationship. Examining the moderating role of FSE serves as a critical lens to understand how personal confidence in using Fintech tools can influence the strength of the relationship between individuals’ positive attitudes toward IUT and their intention to invest. This exploration acknowledges that while positive attitudes are important, the practical ability and confidence to use Fintech platforms can significantly strengthen or reduce the translation of these attitudes into investment actions. This adds a nuanced layer to existing models by highlighting that the effectiveness of attitudes in predicting investment intentions is not uniform but varies according to individual FSE. Such a perspective is particularly relevant in the Malaysian context, where there is a growing intersection of Fintech and Islamic finance, showing that the integration of technological efficiency can be an important factor in the investment decision-making process. This approach deepens our understanding of behavioral finance by incorporating technological dimensions, thus offering a more comprehensive framework in the era of digital finance.

Third, it extended the TPB framework by incorporating IFL. Integrating IFL into the TPB significantly improves the model by adding important dimensions that reflect an understanding of Islamic financial principles and products. This inclusion not only increases the predictive power of the model by providing a deeper understanding of the factors that influence investment intentions, especially knowledge of Islamic principles such as profit sharing and avoiding prohibited businesses, but also increases its cultural and contextual relevance, especially in Malaysia where Islamic finance stands out. Moreover, the integration of IFL into TPB broadens the model’s applicability, making it a useful tool for comparative and cross-cultural studies in behavioral finance related to various religiously compliant financial products. Moreover, it also examined the influence of attitude as a mediator between IFL-intention. This mediating role of attitudes highlights that knowledge alone may not be sufficient to drive behavior. Attitudes act as an important link, translating cognitive understanding into behavioral intentions. The model outlines the psychological process by which education and awareness (IFL) are translated into action (investment intention), emphasizing the importance of not only educating potential investors but also shaping their perceptions and attitudes. It proposes that attitude has a partial mediation effect on investment intention, indicating the dynamic IFL-attitude-intention relationship.

Fourth, it also expanded the TPB framework by examining the direct and indirect effects of religiosity. Although the direct relationship between religious adherence and investment intention in IUT is insignificant, the incorporation of attitudes reveals a subtle pathway. Religious beliefs shape attitudes toward financial products that conform to Islamic principles, and these attitudes, in turn, influence investment intentions. This mediation suggests that the influence of religion on financial decisions operates more through a psychological and value-based lens rather than a simple decision-making process. Understanding these indirect influences is important, especially in a context like Malaysia where religion plays an important role in the cultural and economic landscape, highlighting how deeply embedded beliefs and values can indirectly shape economic behavior.

Regarding managerial implications, understanding the factors that influence the intention to invest in IUT in Malaysia, such as FSE, IFL, religiosity, attitudes and subjective norms, provides important insights for policymakers and funds managers in developing effective marketing and education strategies. Knowledge of these factors enables the creation of user-friendly Fintech platforms, targeted educational initiatives, and marketing strategies that align with Islamic values and foster positive perceptions in IUT. In addition, encouraging a positive attitude towards Islamic financial products can be achieved through promotional efforts by authorities such as the Security Commission and the Malaysian Islamic Development Department (JAKIM), using respected figures as product ambassadors [114]. The marketing strategy should involve not only potential investors but also their social circle, including family and friends, to foster positive behavior towards Islamic financial instruments. This approach recognizes the influence of subjective norms, where those without purchasing power (secondary audience) can influence the decisions of potential investors (primary audience). For example, tech-savvy millennials (secondary audience) can help their parents navigate online investment platforms, combining technological convenience with financial capability.

In addition to fostering positive attitudes through advertising, there is a need for educational programs that explain the advantages and benefits of IUT. Service providers should focus on improving investor access, such as enabling online transactions and ensuring secure platforms, thereby improving service quality and cybersecurity safety features. This effort aims to increase investors’ confidence and trust in online IUT investments. Collectively, these strategies, by leveraging insights from comprehensive behavioral models, assist policymakers, educators and financial institutions in increasing financial literacy and participation in Islamic financial products, thereby promoting a more inclusive and informed financial environment.

## Limitations and suggestions for future studies

As for limitations, the current study provided substantial insights into the factors influencing Malaysians’ IUT investment intention using expanded TPB, but the sample only highlighted Muslim investors. Future studies should extend the research scope to all Malaysians regardless of religion. This study also adopted a cross-sectional research design, thus, future research should utilize a longitudinal and experimental study for a better understanding. The findings of the current study indicated that attitude, subjective norms, IFL, religiosity and FSE explain 43.1 percent of the variance in the intention construct (R^2^ = 0.431). It is feasible that various other drivers, such as personality traits, can be combined to further explain the dependent variable.

## Conclusion

In conclusion, the objective of this study was to contribute to the literature on behavioral finance by investigating the factors that influence investment intentions in IUT among Malaysian investors. To this end, we examined the mediating role of attitude between IFL and religiosity and intention and the moderating role of FSE in the relationship between attitude and investment intention. Building on the TPB, we discovered that attitude, subjective norms, FSE, and IFL directly influence investors’ investment intention, whereas religiosity does not. However, when attitude mediates the relationship, it becomes positive and significant. Moreover, the results indicate that FSE positively moderates the relationship between attitude and investment intention. This study highlights the importance of attitude, IFL, and FSE in investors’ decision-making. It suggests that fund managers and service providers should improve their marketing strategies to enhance investors’ attitudes, IFL knowledge, and fintech skills to attract their interest, confidence, and ability to invest in IUT.

## Supporting information

S1 Appendix (TIF)
