# Supplementary figures and images for: What factors affecting investment decision? The moderating role of fintech self-efficacy
Source: PLoS One. 2024 Apr 18;19(4):e0299004. doi: 10.1371/journal.pone.0299004 (PMC11025860; doi:10.1371/journal.pone.0299004)

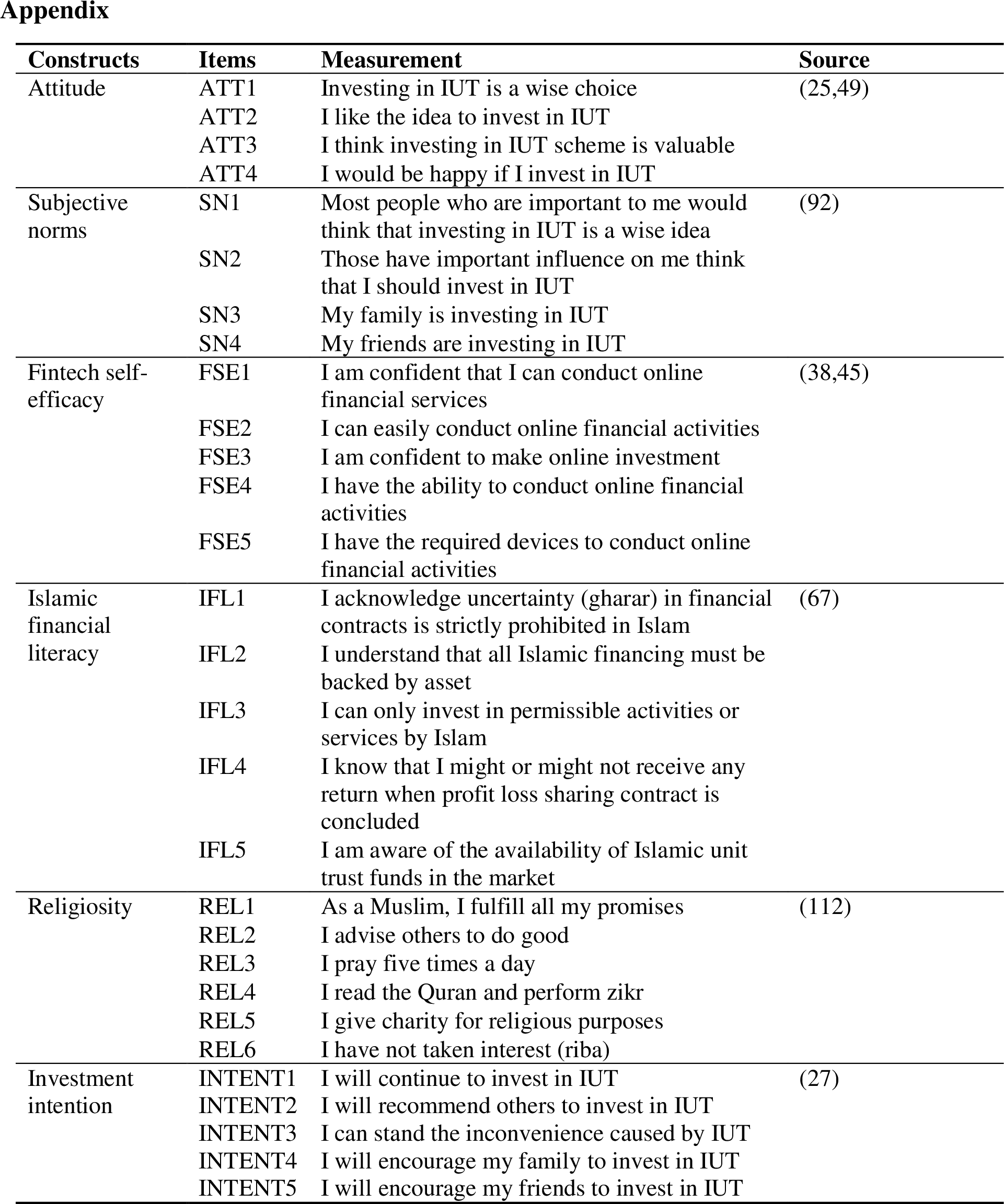

Supplement: S1 Appendix — (TIF) [file pone.0299004.s001.tif]
